# Validity research on ecological momentary assessment in older adults: A scoping review

**DOI:** 10.1093/geroni/igaf122.1792

**Published:** 2025-12-31

**Authors:** Thanakrit Jeamjitvibool, Wirampa Tanglai, Krisada Suamchaiyaphum

**Affiliations:** University of Illinois at Chicago, College of Nursing, Chicago, Illinois, United States; University of Illinois Chicago College of Nursing, Chicago, Illinois, United States; The University of Alabama at Birmingham, Birmingham, Alabama, United States

## Abstract

Ecological momentary assessment (EMA) is a longitudinal self-report method used to assess fluctuating symptoms and health behaviors in real time within individuals’ natural environments during daily activities. Increasing use of technologies such as mobile applications has enhanced EMA research. However, ensuring EMA’s validity and reliability for older populations remains challenging. This study summarized the literature on validity testing of EMA in older adults. We employed the JBI Scoping Review Framework and reporting guidelines. We systematically searched PubMed, Web of Science, CINAHL, PsycInfo, and Embase for relevant articles published through March 10, 2025. Included studies were empirical research published in English and measured behavior or compared EMA to other methods. We excluded review articles, study protocols, and research exclusively utilizing ambulatory assessments without self-reported symptom data. We identified 16 studies, most involving cognitive testing, physical activity, daily activity, depression, pain, alcohol consumption, and fatigue. Most studies (15 of 16) were published after 2016. Sample sizes ranged from 20 to 311 participants. Of the studies, 75% employed mobile applications, while the others utilized Actiwatchs and email. Adherence rates varied between 61% and 95%, with study durations ranging from 5 to 316 days. Leveraging the advantages of EMA design, most studies assessed construct validity, particularly convergent validity, along with criterion and ecological validity. EMA demonstrated satisfactory validity in research involving older adults. The sampling methods and assessment tools used in the studies enhanced EMA’s effectiveness in capturing real-time data on multiple symptoms and health-related behaviors within this population.

